# Search for common genetic variants to allow reliable Mendelian randomization investigations into ketone metabolism

**DOI:** 10.1007/s10654-025-01246-5

**Published:** 2025-06-09

**Authors:** Zhu Liduzi Jiesisibieke, Héléne Toinét Cronjé, C. Mary Schooling, Stephen Burgess

**Affiliations:** 1 School of Public Health, The University of Hong Kong Li Ka Shing Faculty of Medicine, Hong Kong, China; 2 MRC Biostatistics Unit, University of Cambridge, Cambridge, UK; 3 Graduate School of Public Health and Health Policy, City University of New York, New York, USA; 4 Cardiovascular Epidemiology Unit, Department of Public Health and Primary Care, University of Cambridge, Cambridge, UK; 5 Medical Research Council Biostatistics Unit, University of Cambridge, East Forvie Building, Forvie Site, Robinson Way, Cambridge Biomedical Campus, Cambridge, CB2 0SR UK

**Keywords:** Ketones, Genetic variant selection, Cognitive performance, Instrumental variable validity

## Abstract

Ketogenic diets are popular among people aiming for weight management. Ketone supplementation has been linked to improved cognitive performance and increased risk of insulin resistance. We aim to identify common genetic variants that allow Mendelian randomization investigations into further potential effects of ketone metabolism. We set four premises that we believe any valid instrument for ketone metabolism should satisfy. These are: (1) location in a gene region relevant to ketone metabolism, (2) association with all three primary ketone bodies (acetone, acetoacetate, and beta-hydroxybutyrate), (3) no pleiotropic associations, (4) associations with positive control variables (cognitive performance, two-hour glucose, and insulin fold change). We considered gene regions containing variants previously associated with acetone. Four of these regions had biological relevance to ketone metabolism. Lead variants for three of these four regions (*SLC2A4*, *HMGCS2*, *OXCT1*) were associated with all three primary ketone bodies. One region (*SLC2A4*) was associated with two-hour glucose and insulin fold change; however, this region had strong pleiotropic associations with blood pressure. One region (*OXCT1*) showed an association with cognitive performance, and thus satisfied all our premises to be a valid instrument for ketone metabolism. In a complementary agnostic approach considering all genome-wide significant predictors of the three primary ketone bodies in turn, genetically predicted acetoacetate based on seven variants was associated with improved cognitive performance. However, several variants selected in this approach were not located in biologically relevant gene regions and were pleiotropic. Causal claims from Mendelian randomization will be most reliable when the instrumental variable assumptions are plausibly satisfied. We illustrate a framework to identify candidate instruments based on biological considerations.

**Supplementary Information:**

The online version contains supplementary material available at 10.1007/s10654-025-01246-5.

## Introduction

In Mendelian randomization (MR) studies, the selection of suitable genetic instruments for the exposure is a critical factor that determines the validity of causal inferences [1]. When gene regions with biological relevance to the exposure are available, such as regions directly involved in encoding the exposure or traits contributing to its mechanism of action, a biologically-driven strategy for instrument selection is generally preferred as it provides more trustworthy and interpretable results [2]. If this is not possible, then an agnostic, data-driven approach to instrument selection can be performed; however, causal findings from such an approach will only be credible if they are supported by consistent evidence across multiple variants that operate through distinct biological pathways [3].

**Fig. 1:**
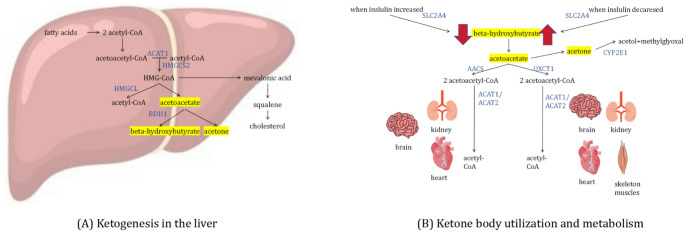
Schematic diagram of biological pathways in the production and metabolism of ketones

This study focuses on ketones—small, water-soluble molecules that play a critical role in energy metabolism, particularly during periods of reduced glucose availability [4, 5]. Ketones are synthesized in the liver from fatty acids [5], a process that involves a series of well-documented biochemical conversions. Initially, fatty acids are transformed into acetoacetyl coenzyme A (acetoacetyl-CoA), which is then converted into β-Hydroxy-β-methylglutaryl coenzyme A (HMG-CoA). HMG-CoA is further metabolized into acetoacetate, a primary ketone body that can be converted to beta-hydroxybutyrate and acetone. The enzymes responsible for these steps are encoded by genes such as *ACAT1*, *HMGCS2*, *HMGCL*, and *BDH1* [6]. In the ketone metabolism pathway, enzymes encoded by *SLC2A4*, *AACS*, *OXCT1*, and *ACAT1*/*ACAT2* are also critical, facilitating the utilization of ketones in organs with high energy demands, including the brain, heart, kidneys, and skeletal muscles (Fig. 1) [5]. Given the well-defined biological pathways of ketone metabolism, employing a biologically driven selection strategy is appropriate in this context.

Ketones are known to support energy metabolism under conditions of low glucose, such as fasting, low-carbohydrate diets, post-exercise states, and neonatal development [5]. Exogeneous ketone usage was found to lower glucose according to a systematic review of trials [7]. Ketones have also been found to associate with how the body uses insulin [8]. Currently exogenous ketone supplements are mainly used by athletes [9] and individuals aiming for weight management [10]. In addition to their metabolic roles, a link between ketones and improved cognitive function has been proposed. Some clinical trials have indicated that ketone supplementation may improve specific components of cognitive function, such as verbal memory and fluency [11–13]. Biological evidence also supports the potential neuroprotective effects of ketones [14], and an MR study has shown that genetically-predicted beta-hydroxybutyrate is positively associated with improved cognitive performance [15].

Existing research has largely focused on individuals with specific health conditions [8, 13, 16] with studies often limited by small sample sizes and short intervention periods [17, 18]. Additionally, the long-term effects of ketones remain unknown. Consequently, there is a need for research that extends beyond clinical populations and short-term interventions to explore the long-term effects of ketones. Potential long-term impacts may include changes in cognitive performance, physical activity levels, and cardiovascular health, including cardiac remodelling [19–21].

Observational studies are frequently compromised by confounding. MR offers an alternative by leveraging genetic variants—typically single nucleotide polymorphisms (SNPs)—as proxies for an exposure [22, 23]. Genetic variants are randomly inherited during meiosis according to Mendel’s laws, and are unaffected by external factors, thus minimizing confounding [24]. Although randomized controlled trials are the gold standard for establishing causality [25], their practical application to investigate long-term ketone effects in healthy populations is limited. In contrast, MR can provide a unique opportunity to examine the causal effect of ketones over the entire lifespan offering insights into therapeutic efficacy and safety that are difficult to capture in time-limited trials [26, 27].

The aim of this investigation is to find genetic variants that are plausibly valid instruments for ketone metabolism. This approach would allow MR investigations to assess the long-term effects of elevated ketone levels on disease outcomes in general populations, by assessing the association of these variants with the disease outcome. In doing this, we hope this study serves as an example of how to select plausible instrumental variables for an MR investigation into the effects of a complex exposure.

## Methods

### Conditions for a valid instrument

An instrumental variable must satisfy three assumptions [28]:

1. Association with the exposure (relevance).
2. No association with the outcome via a confounding pathway (independence).
3. No direct effect on the outcome (exclusion restriction).

These assumptions imply that a valid genetic instrument must affect the exposure in a specific way. Additionally, if we want the genetic variant to provide insight into the impact of an intervention on the risk factor, then the genetic variant must influence the exposure in a way similar to the intervention (this is known as the gene—environment equivalence condition) [24].

Building on this definition, we propose four premises to assess whether the genetic variants are potentially valid instruments based on our understanding of ketone biology:

1. Variants should be located in a gene region encoding a protein that is directly relevant to ketone metabolism (e.g., ketone production or digestion);
2. Variants should be associated with each of the three primary ketone bodies (acetone, acetoacetate, and beta-hydroxybutyrate);
3. Variants should not exhibit horizontal pleiotropy (that is, they should not be associated with risk factors on alternative causal pathways to the outcome);
4. Variants should be associated with traits that have been shown to be downstream effects of ketone supplementation in trials (i.e. positive controls) [29–31]: here cognitive performance, two-hour glucose, and insulin fold change.

### Study design

We considered variants associated with acetone at a p-value < 5 × 10^− 6^ from two independent genome-wide association studies (GWAS): Karjalainen et al. [32] and Borges et al. [33], and assessed whether these variants satisfied the four premises. We used a significance threshold below the standard GWAS threshold to ensure that we did not overlook sub-threshold gene regions with biological relevance to ketone metabolism. A sub-GWAS significant variant in a biologically relevant gene region may be a more valid instrument than a GWAS significant variant whose function is unknown. As a complementary agnostic approach, we took all variants associated with each of the primary ketone bodies in turn and assessed whether these variants gave expected results for the positive control traits.

### Data sources

When multiple GWAS for a given trait were available, we selected the GWAS with the largest sample size and/or most recent data. Information on data sources is shown in Table 1.

**Table 1:** Data sources used in this work

Trait	Data source	Ancestry	Sample size
Acetone	Karjalainen, 2024, Nature	East Asian, South Asian, European	130,311 (4,435 East Asian ancestry individuals, 11,340 South Asian ancestry individuals, 114,536 European ancestry individuals)
Acetone	Borges, 2020, UK Biobank	European	115,075
Acetoacetate	Borges, 2020, UK Biobank	European	115,075
Beta-hydroxybutyrate	Borges, 2020, UK Biobank	European	113,595
Cognitive performance	Lee, 2018, Nature Genetics, SSGAC	European	257,828
Two-hour glucose	Chen, 2021, Nature Genetics, MAGIC	European	63,396
Insulin fold change during an oral glucose tolerance test	Williamson A, 2023, MAGIC	European	53,334

Note: SSGAC: Social Science Genetic Association Consortium; ICBP: International Consortium of Blood Pressure; MAGIC: the Meta-Analyses of Glucose and Insulin-related traits Consortium

### Ketones

Our primary analysis utilized associations from Karjalainen et al. [32], a GWAS meta-analysis of 233 circulating traits, including acetone, which comprised up to 136,016 participants from 33 cohorts. Most samples in this study were serum and were collected after fasting. All studies included in the meta-analysis used NMR-based metabolite quantification, and in each study, traits were standardised to standard deviation units. Most participants (68%) were non-Finnish Europeans. Associations were adjusted for age, sex, principal components and other study-specific covariates. We also utilized associations from Borges et al. [33], which were derived from UK Biobank participants of European ancestry adjusted for age, sex, fasting time, and genotyping chip [33]. Most analysed traits in the UK Biobank were from plasma samples and individuals who were not fasting. For the agnostic strategy, we obtained GWAS summary data from Borges et al. for acetone, acetoacetate, and beta-hydroxybutyrate from the OpenGWAS platform [34], using the following IDs: met-d-Acetone, met-d-Acetoacetate, and met-d-bOHbutyrate [33].

### Cognitive performance

Genetic associations with cognitive performance were obtained from Lee et al. [35], who meta-analysed data from 71 European cohorts, focusing on educational attainment and cognitive phenotypes. The analysis adjusted for age, sex, the interaction between age and sex, and the first 10 principal components. The measure of cognitive performance is described in Trampush et al., 2017 (COGENT consortium) [36] and Davies et al., 2017 (UK Biobank) [37].

### Two-hour glucose

Genetic associations with two-hour glucose (mmol/L) were obtained from Chen et al. [38], who meta-analysed data from participants without diabetes of mixed ancestries. Here we only used the European ancestry subset (*n* = 63,396), which accounts for 74% of the total study population. This GWAS adjusted for body mass index and study-specific covariates.

### Insulin fold change

Genetic associations with insulin fold change (natural logarithm of the ratio between insulin levels at 120 min and baseline) were obtained from Williamson et al. [39], who meta-analysed data on more than 55,000 participants. Here we only used the European ancestry subset (*n* = 53,334) for insulin fold change during an oral glucose tolerance test. This GWAS adjusted for age, sex, population structure, and study-specific covariates.

### Statistical analysis

Associations of variants with primary ketone bodies were assessed based on the lead variant in each gene region. Assessment of pleiotropy was conducted by searching for the gene region in the GWAS Catalogue (https://www.ebi.ac.uk/gwas/). We report all traits associated with a variant at *p* < 5 × 10^− 8^. Associations with positive control traits were assessed based on multiple variants in the gene region (± 200 kilobase pairs) where available, pruning at a threshold of r^2^ < 0.1, using the inverse-variance weighted method. For the agnostic approach, we considered one variant per gene region and performed the inverse-variance weighted, weighted median, and MR-Egger methods [40].

## Results

### Biologically-driven strategy

**Fig. 2:**
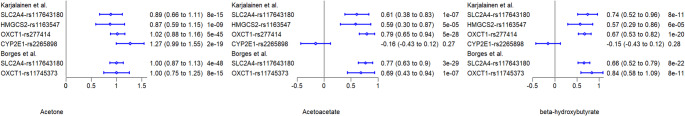
Assessment of genetic associations with all three primary ketone bodies for lead variants in biologically-relevant gene regions. Note: Estimates represent per allele associations of variants with each ketone body in standard deviation units

In the GWAS from Karjalainen et al., 10 gene regions contained variants associated with circulating acetone (Supplementary Table 1). Six of these gene regions had no known clear link to ketone metabolism: *LPL*, *APOA5*, *GALNT2*, *APOC1*, *PPP1R3B*, and *TRIB1*. The remaining four gene regions (based on the nearest gene or the prioritized candidate gene for the lead variant) had known links to ketone metabolism (Fig. 1), and lead variants were associated with acetone at a genome-wide level of significance (*p* < 5 × 10^− 8^). Out of these, lead variants in three gene regions (*SLC2A4*, *HMGCS2*, and *OXCT1*) were associated with all three primary ketone bodies at *p* < 10^− 4^ (Fig. 2), whereas *CYP2E1* was only associated with acetone, and not associated with acetoacetate or beta-hydroxybutyrate (*p* > 0.05).

**Fig. 3:**
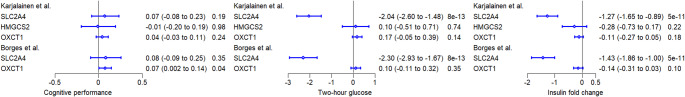
Assessment of genetic associations with positive controls for clumped variants in biologically-relevant gene regions with consistent associations across primary ketone bodies. Note: Estimates represent associations with cognitive performance (standard deviation units), two-hour glucose (mmol/L), and insulin fold change (natural-log of ratio of insulin after 120 min and baseline) per 1 standard deviation increase in genetically-predicted acetone levels calculated using the inverse-variance weighted method (multiple variants) or ratio method (single variant)

After clumping and restricting to genome-wide significant variants, we identified one SNP (rs117643180) for the *SLC2A4* gene region, one SNP (rs1163547) for the *HMGCS2* gene region, and four SNPs (rs6889983, rs277414, rs145006352, rs1155512) for the *OXCT1* gene region. We did not identify statistically significant associations of genetically predicted acetone with cognitive performance for any of these instruments. Higher *SLC2A4*-instrumented acetone levels were associated with lower two-hour glucose and insulin fold change (Fig. 3).

In the UK Biobank GWAS from Borges et al., variants in 40 gene regions were associated with circulating acetone (Supplementary Table 2), of which two encoded proteins with known links to ketone metabolism (*SLC2A4* and *OXCT1*). As above, lead variants in these two gene regions were associated with all three ketone bodies (Fig. 2). We identified the same SNP (rs117643180) for the *SLC2A4* gene region, but a different set of four SNPs (rs62360541, rs115701438, rs11745373, rs6889983) for the *OXCT1* gene region. There was weak evidence for an association of *OXCT1*-instrumented acetone levels with cognitive performance (*p* = 0.04) (Fig. 3). A scatter plot of the variant associations with acetone and cognitive performance shows consistent evidence across these four SNPs in the *OXCT1* gene region, with no clear outliers (Supplementary Fig. 1).

Variants in all three gene regions with consistent associations across ketone bodies (*SLC2A4*, *HMGCS2*, and *OXCT1*) were also associated with other traits (Supplementary Table 3), although some of these associations may reflect upstream or downstream effects of ketones (i.e. vertical pleiotropy) rather than traits on alternative causal pathways to the outcome (i.e. horizontal pleiotropy) [41]. Most notably, the variants in the *SLC2A4* gene region had strong associations with blood pressure, which we judged to reflect horizontal pleiotropy, as changes in blood pressure are not a known consequence of ketone supplementation.

**Fig. 4:**
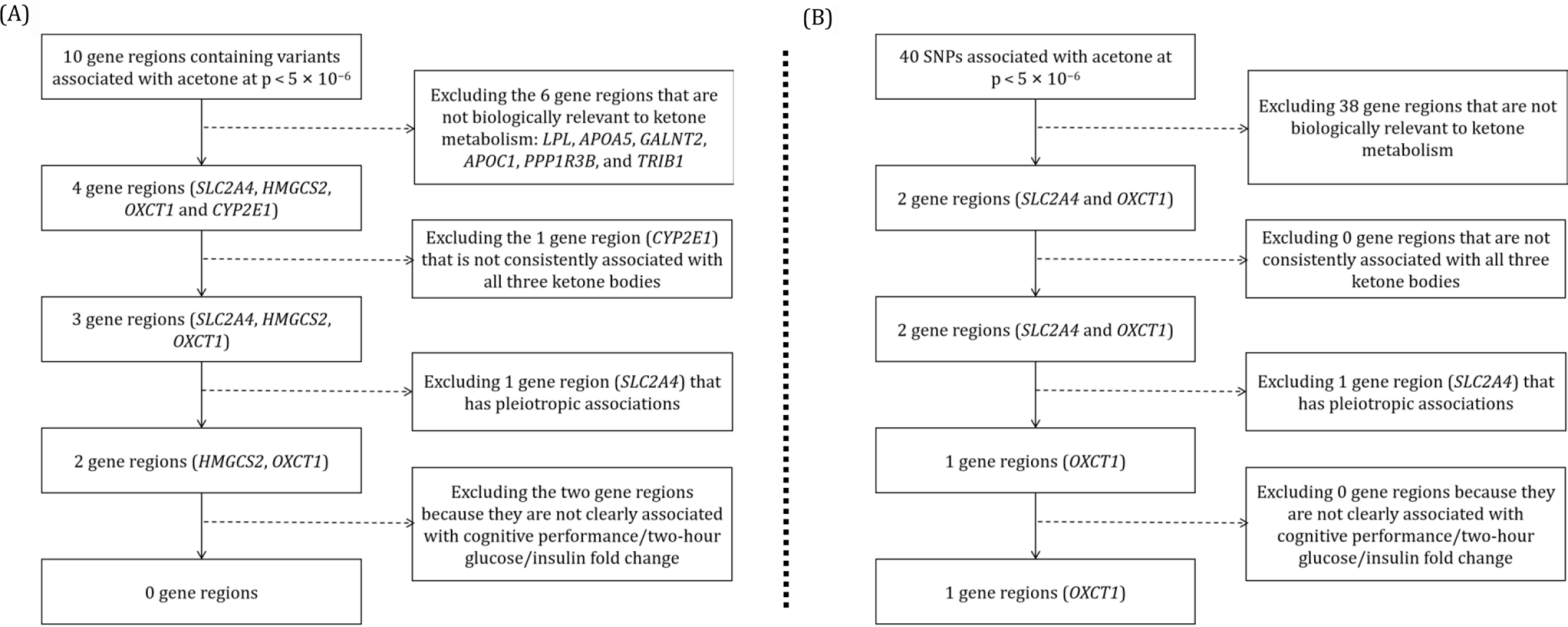
Stepwise search for plausibly valid instrumental variables based on biologically-motivated premises

In summary, only variants in the *OXCT1* gene region chosen based on the Borges et al. GWAS satisfied all four of our premises. A stepwise diagram illustrating our selection strategy is provided as Fig. 4 and a summary of results indicating which variants satisfied each premise is presented as Table 2.

**Table 2:** Summary of assessment of instrumental variable validity using four premises

	Gene name	Lead variant (in bold) and secondary variants after clumping	Premises			
Source			Biological relevance	Consistent for three ketone bodies	No horizontal pleiotropy	Association with at least one positive control
Karjalainen et al.	*CYP2E1*	**rs2265898**	✓	×	-	-
	*SLC2A4*	**rs117643180**	✓	✓	×	✓
	*HMGCS2*	**rs1163547**	✓	✓	✓	×
	*OXCT1*	**rs277414**, rs6889983, rs145006352, rs1155512	✓	✓	✓	×
Borges et al.	*SLC2A4*	**rs117643180**	✓	✓	×	✓
	*OXCT1*	**rs11745373**, rs115701438, rs62360541, rs6889983	✓	✓	✓	✓

Note: We list gene regions with biological relevance to ketone metabolism; full lists of all considered gene regions are given in Supplementary Tables 1 and 2

✓ = premise is satisfied, × = premise is not satisfied, - = premise was not investigated

### Agnostic strategy

**Fig. 5:**
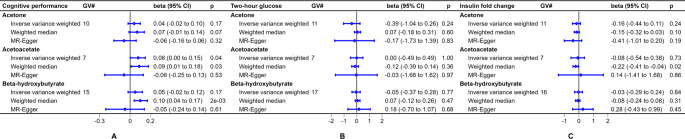
Assessment of genetic associations with positive controls for genome-wide significant genetic predictors of primary ketone bodies in agnostic approach. Note: GV#: the number of genetic variants included in the analysis after clumping and harmonization

Using the GWAS from Borges et al. [33], we identified 12 genome wide significant predictors of acetone, seven genome wide significant predictors of acetoacetate, and 17 genome wide significant predictors of beta-hydroxybutyrate (Supplementary Tables 4–6). We observed a significant positive association of genetically predicted acetoacetate with cognitive performance using the inverse variance weighted method (0.08 standard deviation increase in cognitive performance per 1 standard deviation higher acetoacetate, 95% CI: 0.01 to 0.15, *p* = 0.03) (Fig. 5). In contrast, we did not observe any associations of genetically predicted acetone or beta-hydroxybutyrate with cognitive performance, two-hour glucose, or insulin fold change using the inverse variance weighted method (Fig. 5).

We note that several variants included in these analyses are in gene regions that were (or would have been) excluded from consideration in the biologically driven strategy due to location in gene regions with no clear relevance to ketone biology, inconsistent associations with the three primary ketone bodies, or pleiotropic associations.

## Discussion

In this paper, we proposed four premises as conditions that we would expect to be satisfied by any valid genetic instrument for ketone metabolism. Only variants in the *OXCT1* gene region satisfied all four premises, and even for this gene region, only the variant set based on the UK Biobank GWAS.

It is possible that some of the proposed premises are overly strict. It is not formally necessary for a valid instrument for ketone metabolism to be located in a gene region related to ketone biology. However, we would argue that it is practically unlikely that a variant will be a valid instrument unless this condition holds [2]. Indeed, the pleiotropic nature of variants in several of the gene regions associated with acetone levels in the Karjalainen GWAS [32] that did not encode proteins linked to ketone biology was discussed by the original authors. It is possible that a valid instrument could associate with some but not all primary ketone bodies. However, as they are related molecules on a common biological pathway (Fig. 1), we would argue that any variant that mimics changes in ketone metabolism should be associated with all three primary ketone bodies. It is possible that pleiotropic associations may reflect vertical pleiotropy rather than horizontal pleiotropy. Further, it is possible that the positive control traits are not truly downstream consequences of increased ketone metabolism. However, even if we relax these premises, we would only have four candidate instruments: one of which (*CYP2E1*) only associates with acetone, one of which (*SLC2A4*) has strong associations with blood pressure, leaving two remaining candidate instruments (*HMGCS2*, *OXCT1*).

While it is possible to conduct MR using an agnostic approach for instrument selection, evidence from such an approach will always be less reliable than from a more principled approach. In this case, we did not see convincing and consistent evidence for a causal effect on our control outcomes in our agnostic analyses. The possible exception was for the acetoacetate instrument, which showed some evidence for an association with cognitive performance. However, the association was not strong and would not survive correction for multiple testing.

A previous MR study has been conducted with ketones as the exposure [15]. Sae-jie et al. reported that genetically-predicted beta-hydroxybutyrate was associated with better cognitive performance and lower risk of Alzheimer’s disease [15]. This analysis used five genetic variants in the *DHX38*, *PPP1R3B*, *OXCT1*, *GPAM*, and *TRIB1* gene regions. Investigators also performed a *cis*-MR analysis using only variants in the *OXCT1* gene region, which did not provide evidence of beneficial effects on cognitive performance or Alzheimer’s disease [15]. We would be cautious as to the extent to which the instrumental variable assumptions are satisfied in their primary analysis, and so the extent to which the genetic associations with these outcomes are driven by beta-hydroxybutyrate is uncertain.

Further, even if we accept that some of our genetic variants are valid instruments for ketone metabolism, it is unclear to what extent our analyses would mimic effects of ketone supplementation. Genetic variants specifically associated with blood levels of ketone bodies that act as instruments would allow us to consider the impact of small, life-long variation in endogenous ketone levels, which is not the same as acute ketone elevation resulting from exogenous supplementation or other interventions [42].

We hope this investigation serves as an example of how to select genetic instruments for a complex exposure trait that combines biological knowledge and empirical evidence. While in this case, we were unable to find many suitable instruments, setting clear and testable premises can help identify the most plausible candidate instruments. A further suggestion is to perform MR investigations using variants selected after applying each criterion in a stepwise manner. Investigations based on fewer variants may be less biased, but also have lower power to detect a causal relationship. In practice, analysts typically should present results with different instrument choices, to balance between lack of power to detect a causal effect (due to too strict choice of variants) and lack of specificity in detecting a causal effect (due to too lenient choice of variants). By providing multiple estimates, it may become clear which premises are the key criteria that act as load-bearing pillars for a causal claim from an MR investigation.

There are some limitations of our investigation. Aside from uncertainty about the validity and relevance of the proposed premises, investigations to identify plausible instruments are limited by sample size. It may be that larger GWAS investigations are able to find other predictors of ketone bodies that are valid instruments. Additionally, we have only considered common variants here, as published GWAS typically do not provide estimates for rare variants. It may be that there are rare genetic variants in these or other gene regions that satisfy the instrumental variable assumptions. We did not consider all possible instrument selection criteria here, for example whether our instrument selection was invalidated by selection bias. Finally, our investigations were performed in primarily or solely European ancestry populations. For some exposures, genetic variants that are plausible candidate instruments are only present (or present in much higher frequencies) in non-European populations. Examples include the “Asian flushing” variant in the *ALDH2* gene [43], and common predictors of lipoprotein-associated phospholipase A2 in the *PLA2G7* gene in South and East Asians [44].

In conclusion, choice of genetic variants is critical to the validity of a MR investigation. We have implemented a systematic framework to identify valid genetic instruments for ketone metabolism. While we did identify variants in one gene region that satisfied all the desired premises, the evidence for instrument validity is still questionable. Therefore, it may not be possible to perform reliable MR investigations to learn about the effects of increasing ketone metabolism. Some epidemiological questions cannot be answered reliably using the MR framework. We hope this study can help researchers critically reflect on how to select reasonable genetic variants when performing MR studies for complex exposures.

## Electronic supplementary material

Below is the link to the electronic supplementary material.

Supplementary Material 1

Supplementary Material 2

## Data Availability

This study only used publicly available data.
