# Supplementary figures and images for: Search for common genetic variants to allow reliable Mendelian randomization investigations into ketone metabolism
Source: Eur J Epidemiol. 2025 Jun 9;40(6):649–57. doi: 10.1007/s10654-025-01246-5 (PMC12263766; doi:10.1007/s10654-025-01246-5)

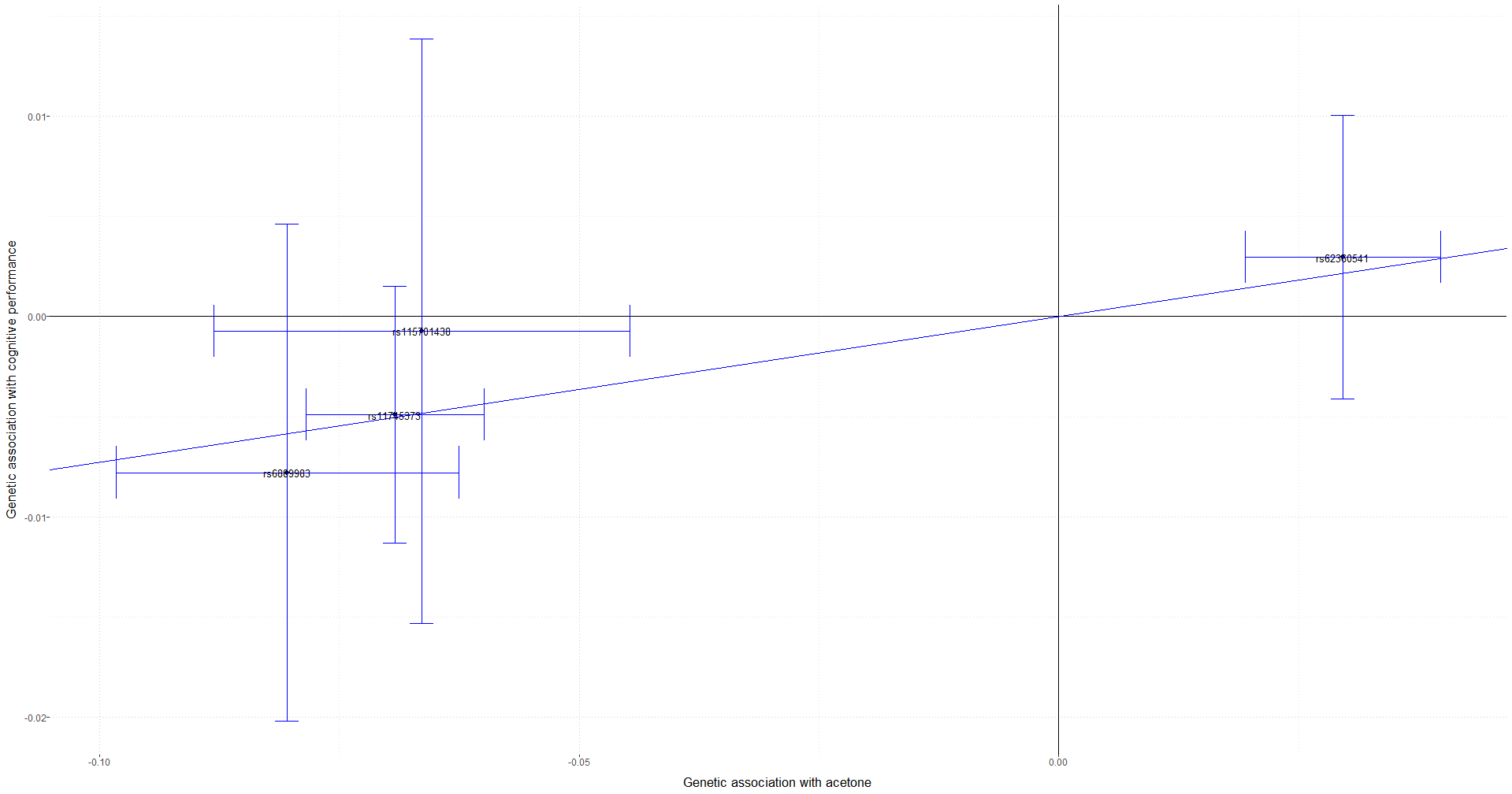

Supplement: Supplementary file 1 — Supplementary Material 1 [file 10654_2025_1246_MOESM1_ESM.jpg]
